# Shuangshi Tonglin capsule improves chronic prostatitis through the SIRT-1/AMPK and MAPK signalling pathways

**DOI:** 10.1016/j.heliyon.2023.e21745

**Published:** 2023-11-04

**Authors:** Qing Liu, Xinyue Zhang, Peng Mao, Ziqiang Wang, Qian Mao, Chuan Wang, Jiping Liu, Xingmei Zhu, Baoan Wang, Hao Wei

**Affiliations:** a School of Pharmacy, Shaanxi University of Chinese Medicine, Xianyang, 712046, Shaanxi, China; b Shaanxi Momentum Pharmaceutical Co., Ltd., Xianyang, 712000, Shaanxi, China

**Keywords:** Shuangshi Tonglin capsule, Chronic prostatitis, Inflammation, Oxidative stress, AMPK/SIRT-1, MAPK

## Abstract

**Objectives:**

To explore the effects of the Shuangshi Tonglin (SSTL) capsule on CP/CPPS and reveal the therapeutic mechanisms.

**Methods:**

A CP/CPPS rat-model group received an intraprostatic injection of CFA. SSTL capsule were administered daily by oral gavage at doses of 1.25, 2.5, and 5.0 g/kg for 28 days. Pain threshold tests were performed, and prostate and blood samples were collected. We performed histological analysis of the prostate tissue and immunohistochemical analysis of TNF-α and COX-2. Measure the TNF-α levels, detect antioxidant levels in serum and prostate tissue, and evaluate the expression of proteins with the AMPK/SIRT-1 and MAPK signalling pathways.

**Results:**

After SSTL capsule treatment, all animals exhibited an increased mechanical pain threshold in the lower abdomen, decreased inflammation in the stroma, and reduced histological structural damage. Inflammation was reduced through the observed decrease in the levels of various inflammatory factors, as well as in the increase of the levels of MDA, *p*-AMPK, and SIRT-1. The suppression of IKKβ, p-P38, *p*-ERK and *p*-JNK was also observed.

**Conclusions:**

SSTL capsule treatment decreased inflammation in the stroma and reduced histological structural damage. It improved CP/CPPS symptoms by inhibiting oxidative stress and inflammation. Our study indicates that the SSTL capsule is an effective treatment for prostatitis.

## Graphical abstract

**Figure:**
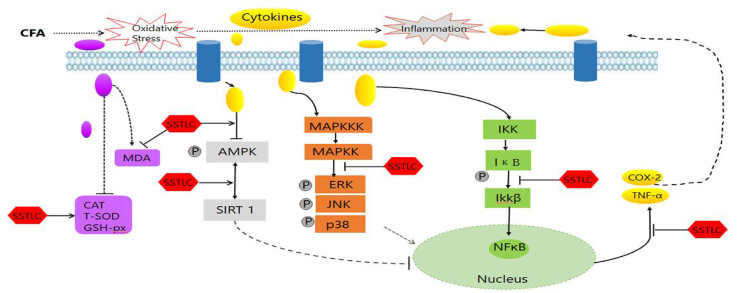

## 1 Introduction

Prostatitis is a common disease in the urinary system affecting men of all ages, although it primarily occurs in middle-aged men [1]. Chronic prostatitis (CP) with chronic pelvic pain syndrome (CPPS) is one of the four categories of prostatitis, and its prevalence is over 90–95 % within prostatitis cases. CP/CPPS affects the quality of life of 10–15 % of men [2,3], making it a prevalent health problem. However, while standard treatments such as antibiotics, α-receptor blockers, anti-inflammatory drugs, plant therapy, hormone therapy, and other therapies are likely to result in a decrease in prostatitis symptoms, there are often side effects with long-term use of these drugs. The aetiology, pathology and physiological changes of CP/CPPS are not well understood. Most scholars believe that the main cause of CP/CPPS may be multiple, converging pathogenic factors [4]. Therefore, it is imperative for researchers to find a new and effective methods for the treatment of CP/CPPS.

Shuangshi Tongling Capsule is a traditional Chinese medicine compound preparation that has been approved and marketed in China and is widely used in clinical practice (CFDA approval number No. Z20080028). it composed of 10 traditional Chinese medicinal and mineral compounds, including, *Dioscorea hypoglauca* Palib*., Phellodendron amurense* Rupr*., Patrinia scabiosifolia* Link*., Strobilanthes cusia* (Nees) Kuntze*.,* Talcum*, Plantago asiatica* L*., Acorus tatarinowii* Schott*, Poria cocos* (Schw.) Wolf*, Atractylodes lancea* (Thunb.) DC*.,*and *Salvia miltiorrhiza* Bunge*.* One clinical study demonstrated that SSTL capsule treatment improved the quality of life of patients, indicating potential therapeutic effects on CP/CPPS [5,6]. Some traditional Chinese medicine compounds in SSTL capsule inhibit oxidative stress and inflammation [7]. Phellodendri Cortex has many herapeutic effects which include anti-inflammatory, antioxidant. Traditionally, its medicinal part could exert therapeutic effects in various diseases such as pneumonia [8]. Phellodendri Cortex has anti-inflammatory effects, via inhibition of NO production，reduce of pro-inflammatory cytokines [9]. Dioscin is a typical saponin with multiple pharmacological activities. Such as antitumor, antimicrobial, anti-inflammatory, antioxidative [10]. Patrinia scabiosifolia is a commonly used Chinese medicinal plant, which it has anti-inflammatory activities [11]. Salvia miltiorrhiza Bge. is widely applied in the treatment of systemic diseases. Research has shown that compounds isolated from Salvia miltiorrhiza Bge. have anti-inflammatory, anti-oxidative activities [12].

Traditional Chinese medicine compounds are multichannel, multidirectional, and exhibit low toxicity and minimal side effects in the treatment of diseases. SSTL capsule have been used in the treatment of CP/CPPS; however, their mechanism of treatment is not clear. Thus, the goal of this study is to explore and clarify the mechanisms by which SSTL capsule aid in the treatment of CP/CPPS.

## 2 Methods and materials

### 2.1 Materials

SSTL capsule were obtained from Shaanxi Momentum Pharmaceutical Co., Ltd. (Shaanxi, China). Tamsulosin Hydrochloride Sustained Release Capsule (TH) was obtained from Astellai Pharmaceutical Co., Ltd (Shenyang, China). Complete Freund's adjuvant (CFA) was obtained from Sigma-Aldrich, Inc (St. Louis, USA). Sodium carboxymethyl cellulose (CMC-Na) was purchased from Merck & Co., Inc. (Whitehouse Station, USA).

### 2.2 Sample preparation

Precision 1 g of the contents of the SSTL capsule was weighed and placed in a 50 mL volumetric flask. Approximately 45 mL of a mixed solution of hydrochloric acid-methanol (1:100) was added, and the mixture was ultrasonically treated (250 W, 40 kHz) for 45min, brought up to the mark with acid-methanol, shaken well, filtered, precisely pipetted 5 mL into a 10 mL volumetric flask, diluted to volume, shaken well, filter through a 0.22 μm microporous membrane, and used as the test solution.

### 2.3 HPLC analysis of berberine hydrochloride in SSTL capsule

The SSTL capsule was analyzed by LC-2010C series HPLC, chromatographic column is SB-C_18_ column (Agilent, 4.6 mm × 250 mm; 5 μm). The flow rate was set at 1.0 mL/min. The amount of injection was 10 μL. The acetonitrile (A) and water (B) (48：52) (Per 1000 mL containing potassium dihydrogen phosphate 3.4 g, sodium dodecyl sulfonate 1.2 g) were selected as the mobile phase. The detection wavelength was 270 nm. The theoretical plates number according to the peak calculation of Berberine hydrochloride not be less than 5000.

### 2.4 Animal model

Male Sprague–Dawley (SD) rats (200–250 g) were purchased from Chengdu Dashuo Experimental Animal Co., Ltd. The animals were housed in Shaanxi University of Traditional Chinese Medicine at a temperature of 16–25 °C, and food and water were provided ad libitum. The animal experiments were approved by the animal ethics committee of Shaanxi University of Traditional Chinese Medicine (SUCMDL2019401001).

### 2.5 Experimental method

The CP/CPPS rat model was established by intraprostatic injection of CFA. The prostates of anaesthetized rats were exposed through an incision in the lower abdomen. For the CP/CPPS [13] rats, 0.1 mL CFA was injected into the prostate, and for the sham group, 0.1 mL normal saline was injected into the prostate. A total of 72 SD rats were divided into six groups of 12 SD rats each.The clinical dosage of SSTL for human is 2 g each time, three times a day, a total of 6 g per day, according to the conversion of human and rat experiments, the dose conversion factor for human is about 36, and the dose conversion factor for rat is about 6.25, and the body weight of SD rats selected for the experiments is about 250 g. According to the conversion formula, the equivalent dose for rats is 1.25 g/kg/d, the medium dose is twice the human dose of 2.5 g/kg/d, and the high dose is four times the human dose of 5.0 g/kg/d. All animals were treated for 28 days as follows: 1) Sham group: 0.5 % CMC-Na; 2) CP/CPPS model group: 0.5 % CMC-Na; and 3) Low-dose group: 1.25 g/kg/d SSTL capsule; 4) Middle-dose group: 2.5 g/kg/d SSTL capsule; 5) High-dose group: 5.0 g/kg/d SSTL capsule; 6) Tamsulosin Hydrochloride Sustained Release Capsule (TH) group: 0.0208 g/kg/d TH capsule. SSTL capsule and TH were dissolved in 0.5 % CMC-Na in every group. The oral dose for the rats was 1 mL/100 g/d.

After 28 days of treatment, the rats were anaesthetized. Abdominal aortic blood sampling and dissected prostate tissue were collected. The prostate tissue samples were fixed in a 4 % paraformaldehyde solution, and other prostate tissue was placed in cryovials and immediately placed in liquid nitrogen for storage.

### 2.6 Pain threshold test

Rats were tested on days 1 and 35 via application of Von Frey hair to the lower abdomen to evaluate mechanical pain thresholds. According to the pre-test, the selected measured Von Frey hairs were 0.04, 0.07, 0.16, 0.4, 0.6, 1, 1.4, and 2 (g). When measuring, the median value of 0.4 was selected as the starting point and measured in ascending order. When the first crossover occurred, the next four occurrences were then measured. The log unit value corresponding to the last measured Von Frey hair was substituted into the formula.

The von Frey hair was bent into an “s" shape for 6–8 s, with an interval of at least 7 s between two adjacent stimuli. Rats moving their abdomen or licking the stimulated side was considered to be a positive reaction. In the process of stimulation, rats walking or licking their body as soon as they were stimulated were considered likely positive. A positive result was recorded as X, and a negative result was recorded as O.

Our calculations used the following formula: 50%g threshold=(10^[Xf + kδ]^)/10000.

The XF = G number corresponding to the last measurement was logarithmic, the K value was derived from the reference table [14], δ = 0.267 (as mentioned above), and the final result of the formula was the G number.

### 2.7 Prostate index

The prostate samples were collected and accurately weighted. Prostate index = prostate weight (mg)/body weight (g) was the formula used for analysis.

### 2.8 Hematoxylin and eosin (H&E) staining

The prostate tissues were fixed in 4 % paraformaldehyde solution, the fixed prostate tissues were trimmed flat, dehydrated in gradient alcohol, the dehydrated prostate tissues were embedded in paraffin wax, cut into sections with a thickness of 5 μm, and the sections were dewaxed (put into xylene, anhydrous ethanol, and 75 % alcohol in the order of the sections), and then stained with hematoxylin staining, differentiated, counterblue, dehydrated, and stained with eosin staining, and then finally sealed. The whole slide image analysis was performed by the APERIO AT2 system (Leica Biosystems, Wetzlar, Germany).

### 2.9 Immunohistochemistry analysis

Immunohistochemistry (IHC) analysis was used to detect the expression of TNF-α and COX-2 in prostate tissue. The prostate tissue sections were dewaxed in water, and the sections were washed with PBS (pH = 7.4). After washing, the tissue sections were treated with 3 % H_2_O_2_ solution for 25 min at room temperature. After blocking, the sections were incubated overnight at 4 °C. Then, AEC colour solution and hematoxylin were used for redyeing. Five randomly distributed fields within the prostate lobe on each slide were analyzed, and cells with red granules were positive. Each slide was selected for TNF-α- and COX-2- positive cell counting.

### 2.10 Analysis of TNF-α levels in serum and tissue

The ELISA method was used to determine the level of the inflammatory cytokine TNF-α in serum and prostate tissue according to the manufacturer's instructions (Shanghai Enzyme Biotechnology Co., Ltd. Shanghai, China).

### 2.11 Assay for antioxidant markers in serum and prostate

The serum and prostate activities of antioxidants MDA, T-SOD, CAT, and GSH-PX were evaluated by using ELISA kits (Nanjing Jiancheng Bioengineering Institute. Nanjing, China), according to the manufacturer's instructions.

### 2.12 Western blot analysis

Total proteins were extracted from prostate tissues with RIPA buffer. Equal amounts of protein samples were loaded onto 10 % SDS-PAGE gels and then transferred onto PVDF membranes. The PVDF membranes were blocked with 5 % non-fat milk for 2 h at room temperature and then incubated with anti-IKKβ (1:1000 dilution, CST), anti-P38 (1:1000 dilution, CST), anti-phospho-P38 (1:1000, dilution, CST), anti-ERK1/2 (1:1000 dilution, CST), anti-phospho-ERK1/2 (1:1000 dilution, CST), anti-JNK/SAPK (1:1000 dilution, CST), anti-phospho-JNK/SAPK (1:1000 dilution, CST), anti-AMPK (1:1000 dilution, CST), anti-phospho-AMPK (1:1000 dilution, CST), anti-SIRT-1 (1:1000 dilution, Proteintech), and anti-GAPDH (1:1000 dilution, BOSTER) overnight at 4 °C. The next day, the PVDF membranes were incubated with a secondary antibody for 2 h at room temperature. The protein signals were visualized with an enhanced chemiluminescence system and detected by ImageJ software. All protein signals were standardized by using GAPDH.

### 2.13 Statistical analysis

All the data were analyzed with SPSS 24.0, and the results are expressed as the mean ± standard error of the mean (mean ± SEM). Multiple comparisons were performed using one-way analysis of variance (ANOVA), and P < 0.05 was considered statistically significant. Statistical analyses were performed using GraphPad Prism 8.0 software (GraphPad Software, Inc., CA, USA).

## 3 Results

### 3.1 HPLC analysis of berberine hydrochloride in SSTL capsule

**Fig. 1:**
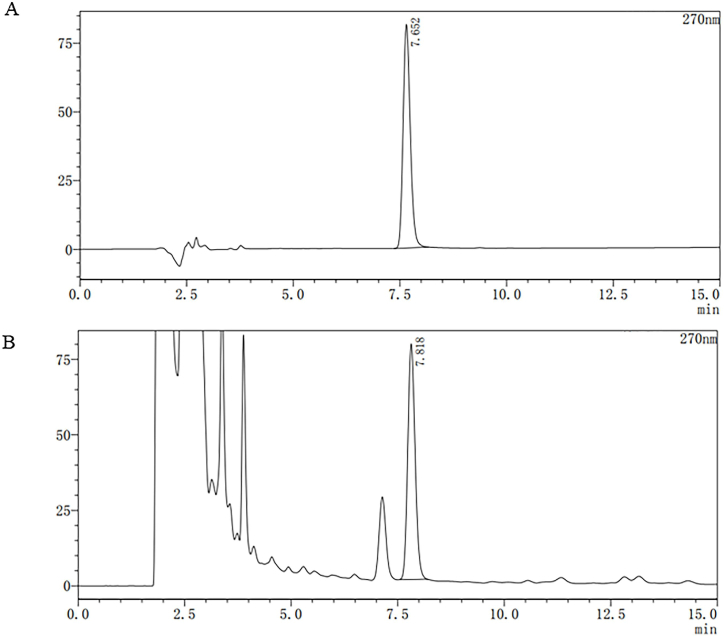
HPLC analysis of SSTL capsule. (A) The Standard. (n = 2) (B) The samples of SSTL capsule. (n = 6).

For quantitative analysis of berberine hydrochloride in the SSTL capsule, the retention time of the reference substance was 7.625 min, and the retention time of the sample was 7.818 min. Representative HPLC chromatograms of the reference standard and SSTL capsule extracts were generated (Fig. 1).

### 3.2 Effects of SSTL capsule on mechanical pain threshold, prostate index and body weight

**Fig. 2:**
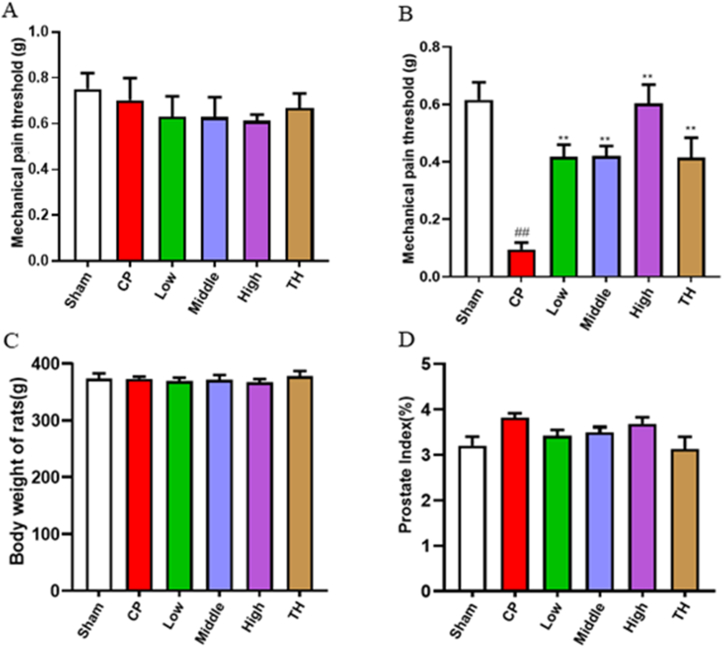
Mechanical pain threshold，body weight and prostate index. (A) Before modeling; (n = 6) (B) After 28 d of treatment; (n = 6) (C) Body weight; (n = 6) (D) Prostate index. (n = 6) Values are presented as means ± SEMs. ^#^P < 0.05, ^##^P < 0.01 compared with the Sham group; *P < 0.05, **P < 0.01 compared the CP group.

In the CP/CPPS rats, before the injection of CFA into the prostate, mechanical pain thresholds of the lower abdomen were not significant (Fig. 2A). After 28 days of treatment, all animals had a significantly increased mechanical pain thresholds in the lower abdomen (P < 0.01) (Fig. 2B).

Compared with the CP/CPPS group, the high, middle, and low dose SSTL capsule groups and the TH group showed no significant differences in body weight or prostate index (Fig. 2C and D).

### 3.3 Effects of SSTL capsule on prostate histopathology

**Fig. 3:**
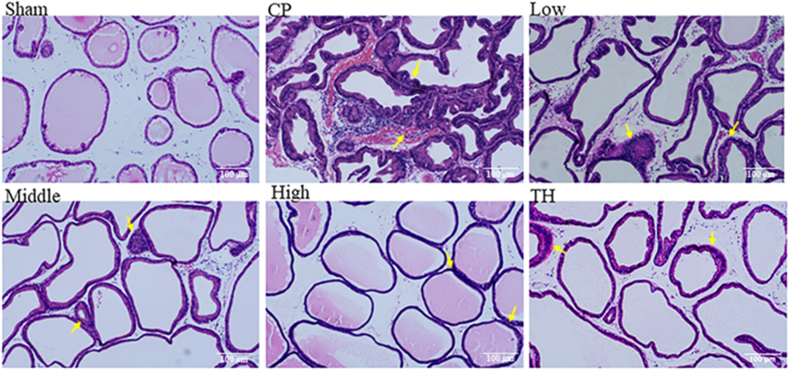
Photomicrographs of H&E stained of rat prostate tissue (100 × )(n = 6).

Compared with the sham group, the CP/CPPS group showed obvious histological structural damage, increased inflammation in the stroma, and irregularly sized prostate cells (Fig. 3). Compared with the CP/CPPS group, the high, middle, and low dose SSTL capsule and TH groups showed decreased inflammation in the stroma and reduced histological structure damage. In addition, the high-dose SSTL capsule and TH groups showed no significant inflammation.

### 3.4 Effect of SSTL capsule on prostate IHC staining of TNF-α and COX-2

**Fig. 4:**
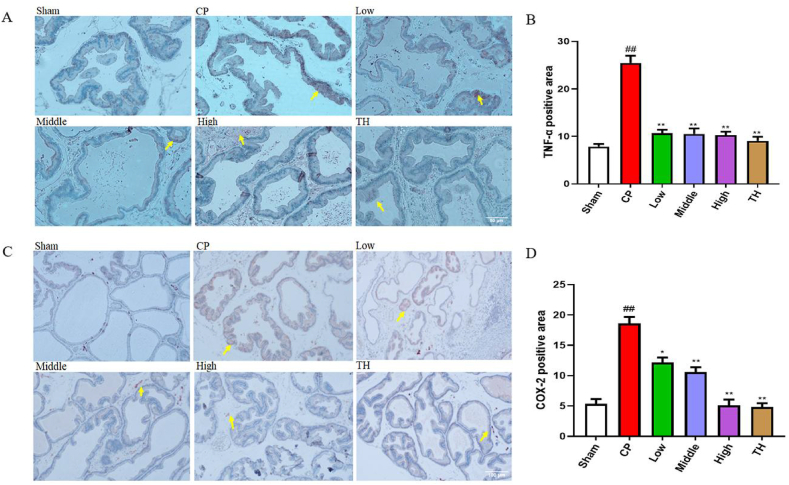
Effect of SSTL capsule on COX-2 in prostate tissue (200 × ). (A) Photomicrographs of immunohistochemical for the effects of SSTL capsule on the expressions of TNF-α, the yellow arrows indicate the expression of TNF-α; (n = 6) (B) TNF-α positive area. (n = 6) (C) Photomicrographs of immunohistochemical for the effects of SSTL capsule on the expressions of COX-2, the yellow arrows indicate the expression of COX-2; (n = 6) (D) COX-2 positive area. (n = 6) Values are presented as the means ± SEMs. ^#^P < 0.05, ^##^P < 0.01 compared with the Sham group; *P < 0.05, **P < 0.01 compared the CP group. Values are presented as the means ± SEMs. ^#^P < 0.05, ^##^P < 0.01 compared with the Sham group; *P < 0.05, **P < 0.01 compared the CP group.

The expression levels of TNF-α and COX-2 were evaluated through immunohistochemical analysis of prostate tissue. In (Fig. 4A and B) yellow arrows indicate the expression of TNF-α. Compared with the sham group, the CP/CPPS group showed an increased expression of TNF-α. Compared with the CP/CPPS group, the high, middle, and low dose SSTL capsule and TH groups showed an expression of COX-2 that was significantly reduced (P < 0.01). In (Fig. 4C and D) the yellow arrows indicate the expression of COX-2. Compared with the sham group, the CP/CPPS group showed increased COX-2 expression. Compared with the CP/CPPS group, the expression of COX-2 was reduced following SSTL capsule and TH treatment. In particular, the expression of COX-2 was significantly reduced in the high-dose SSTL capsule and TH groups.

### 3.5 Effects of SSTL capsule on serum and tissue TNF-α levels

**Fig. 5:**
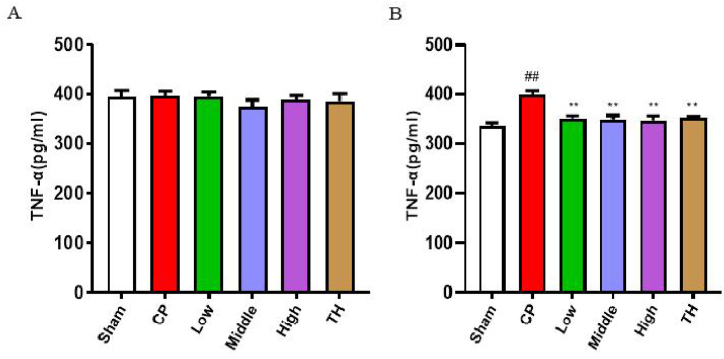
The expression levels of TNF-a in serum and prostate tissue. (A) Serum; (n = 6) (B) Tissue. (n = 6)Values are presented as the means ± SEMs. ^#^P < 0.05, ^##^P < 0.01 with the Sham group; *P < 0.05, **P < 0.01 with the CP group.

Since inflammatory mediators are direct markers of inflammation, we measured TNF-α to assess the degree of inflammation between different groups (Fig. 5). Compared with the sham group, the CP/CPPS group showed that the prostate tissue proinflammatory cytokine TNF-α was significantly increased (P < 0.01). After SSTL capsule and TH treatment, serum TNF-α levels were significantly reduced in the sham group compared with those of the CP/CPPS group. However, the serum levels of TNF-α were not significantly different (P > 0.05).

### 3.6 Effects of SSTL capsule on antioxidant capacity and suppressed oxidative stress

Compared with the Sham group, the CP/CPPS group showed that serum levels of CAT, T-SODD, and GSH-PX were significantly decreased (P < 0.01). After SSTL capsule treatment, the CAT, T-SOD, and GSH-PX levels in serum significantly increased in the sham group compared with those of the CP/CPPS group (P < 0.05), while the levels of MDA were not significantly changed (P > 0.05).

**Fig. 6:**
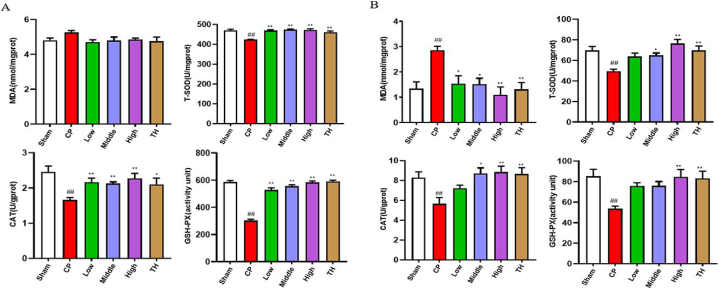
Effect of SSTLC on antioxidant capacity and suppressed oxidative stress. (A) Serum; (n = 6) (B) Tissue. (n = 6) Values are presented as the means ± SEMs. ^#^P < 0.05, ^##^P < 0.01 with the Sham group; *P < 0.05, **P < 0.01 with the CP group.

Compared with the sham group, the CP/CPPS group showed significantly decreased CAT, T-SOD, and GSH-PX levels in the prostate tissue, and significantly increased MDA levels (P < 0.01). Compared with the CP/CPPS group, the low, middle, and high dose SSTL capsule and TH groups showed that MDA decreased significantly. The middle and high dose SSTL capsule and TH groups showed significant decreases in CAT and T-SOD levels, and the high dose SSTL capsule and TH groups showed significant increases in GSH-PX (P < 0.05 or P < 0.01). In the low dose SSTL capsule group, changes in CAT, T-SOD and GSH-PX levels were not significant (P > 0.05) (Fig. 6).

### 3.7 Effects of SSTL capsule on inflammatory pathways

**Fig. 7:**
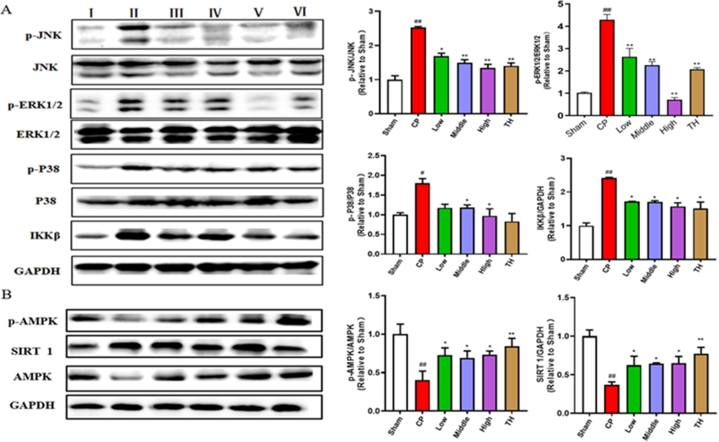
Effect of SSTL capsule on MAPKs and SIRT 1/AMPK signaling pathway. (A) Western blot analysis of MAPK signaling pathway, IKKβ and GAPDH expression in prostate tissues; (n = 3) (B) Western blot analysis of SIRT 1, *p*-AMPK, STAT-3 and GAPDH expression in prostate tissues. (n = 3) Ⅰ: Sham group Ⅱ: CP group Ⅲ: Low group Ⅳ: Middle group Ⅴ: High group Ⅵ: TH group. Values are presented as the means ± SEMs. ^#^P < 0.05, ^##^P < 0.01 with the Sham group; *P < 0.05, **P < 0.01 with the CP group.

To explore the therapeutic mechanism of action of SSTL capsule on CP/CPPS, Western blotting was used to analyse proteins from the MAPKS and AMPK signalling pathways, IKKβ and SIRT-1 (Fig. 7). Compared with the sham group, the CP/CPPS group showed increased expression of IKKβ, p-P38, *p*-ERK, and *p*-JNK, and reduced expression of *p*-AMPK and SIRT-1. After treatment with SSTL capsule, the expression levels of IKKβ, p-P38, *p*-ERK, and *p*-JNK were significantly suppressed in a dose-dependent manner, and the expression levels of *p*-AMPK and SIRT-1 were increased (P < 0.05 or P < 0.01).

## 4 Discussion

The SSTL capsule was developed through the “Chengshi Bixie fenqing Decoction” by Cheng Zhongling, a famous healer of the Qing Dynasty. The SSTL capsule is authorized in China to treat CP/CPPS. The literature has proven that SSTL capsule have significant anti-inflammatory, analgesic, and diuretic effects [15], suggesting that SSTL capsule may be beneficial in CP therapy. However, its therapeutic mechanism of action in CP/CPPS treatment is not understood.

Studies have demonstrated that CP/CPPS has a complex pathophysiology, and its aetiology is unclear, though autoimmune and inflammatory processes are thought to have major roles in the development of CP/CPPS. The establishment of a reliable animal model of CP/CPPS is an important step toward understanding the disease. CFA-induced CP/CPPS rats showed obvious histological structural damage in prostate tissue, increased inflammation in the stroma, and irregularly sized prostate cells [16]. Our present study indicated that the SSTL capsule decreased inflammation in the stroma, reduced histological structure damage, decreased the delivery of COX-2 and TNF-α in prostate tissues, decreased the expression of the proinflammatory cytokine TNF-α, improved the levels of CAT, T-SOD and GSH-PX, and reduced the levels of MDA. Regarding the influence of inflammatory signalling pathways on CP/CPPS, the results demonstrated that treatment with SSTL capsule suppressed the expression of IKKβ, p-P38, *p*-ERK, and *p*-JNK, and increased the expression of *p*-AMPK and SIRT-1. This indicated that the SSTL capsule may improve CP/CPPS symptoms by inhibiting oxidative stress and inflammation.

Inflammatory mediators are crucial immunoregulatory albumens that play a role in adjusting physiological parameters [17]. The aberrant expression of inflammatory mediators can lead to a reduced activated immune reaction, which has been reported in inflammatory illness [18]. Proinflammatory mediators such as TNF-α and IL-1β are among the proximal risk factors for CP/CPPS. TNF-α is synthesized by monocytes and macrophages and plays a key role in phlogistic sickness. Multiple clinical studies have found that TNF-α is higher in CP/CPPS patients [19,20]. One study found that damp-heat type CP/CPPS was correlated with the levels of the proinflammatory cytokines TNF-α and IL-1β in prostate fluid [21]. In this study, immunohistochemical staining and ELISA results showed that, compared with the sham group, the CP/CPPS group displayed significant increases in proinflammatory cytokine TNF-α in prostate tissue increased. After SSTL capsule treatment, TNF-α expression was reduced. This result indicated that the SSTL capsule treats CP/CPPS by reducing inflammatory mediators.

In addition, oxidative stress is one of the main mechanisms in CP/CPPS pathology. This study demonstrates the involvement of cytokine-mediated inflammation, a key component in oxidative stress. This and other studies indicate that we can treat CP/CPPS with antioxidants. MDA is a lipid peroxidation product that is frequently considered a biomarker of oxidative injury [22]. In contrast, T-SOD is the first killer of oxygen free radicals in the human body. Under the action of enzymes, superoxide free radicals are converted into hydrogen peroxide and water through disproportionation reactions, and hydrogen peroxide generated by T-SOD can be converted into water by GSH-PX and CAT [23]. In this study, our results show that the CFA-induced CP/CPPS is associated with decreased antioxidant enzymes, and improved lipid peroxidation products may have the capability to enhance antioxidant activity.

Several experiments have shown that MAPK and NF-κ B play key roles in the development of CP/CPPS [[23], [24], [25]]. MAPKs are important for inflammatory signalling pathways, since they accept stimulant semaphores and transfer them through the phosphatase activation cascade. P38, ERK, and JNK, which represent subfamilies of MAPK, are activated by proinflammatory cytokines and oxidative stress and induce the phosphorylation of many significant signalling molecules [26]. Proinflammatory cytokines and oxidative stress are the main factors that lead to the phosphorylation of p38, ERK1/2, and JNK and further promote the production of inflammatory cytokines and aggravate of oxidative stress.

IKKβ, for example, can activate the NF-κ B signalling pathway. At rest, NF-κ B binds to IKKβ to form a trimer. However, when cells are irritated by inflammation, IKKβ is degraded by proteases, and p65 is translocated to the nucleus to adjust transcription levels, encode proinflammatory mediators, and exacerbate inflammation [26,27]. Inflammation is associated with the AMPK pathway and SIRT-1. AMPK levels in this study were positively correlated with SIRT-1 levels. The inflammation inhibited by the AMPK pathway can be activated by SIRT-1 [28]. Studies have shown that AMPK can indirectly or directly inhibit the activity of NF-κ B by downstream SIRT-1, FOXO3a, p53 and other proteins, thereby inhibiting the expression of inflammatory factors [[29], [30], [31]]. Current studies indicate that AMPK may play an anti-inflammatory role through different MAPK subfamilies [[32], [33], [34]]. In our experiment, SSTL capsule obviously suppressed the expression of IKKβ, p-P38, *p*-ERK, and *p*-JNK, and increased the expression of *p*-AMPK and SIRT-1. The results showed that SSTL capsule reduced inflammation by activating the SIRT-1/AMPK signalling pathway and inhibiting the MAPK signalling pathways.

## 5 Conclusion

In summary, traditional Chinese medicine has been important in treating many challenging diseases.

The development of novel and successful treatments for CP/CPPS and other diseases requires further research. The purpose of this study was to explore the effects of SSTL capsule on CP/CPPS in an animal model, and the study did in fact generate novel data in a CP/CPPS animal model demonstrating that the SSTL capsule alleviated pain by altering the inflammatory pathways in CP/CPPS through the targeting of the MAPK pathway and the SIRT-1/AMPK pathway. Based on the experimental results, our study shows that the SSTL capsule is an effective treatment for prostatitis.

## Author contribution statement

Qing Liu, Chuan Wang and Hao Wei were responsible for the design of the study. Xinyue Zhang, Qian Mao and Ziqiang Wang performed the experiment and the statistical analysis and wrote the manuscript. Jiping Liu and Xingmei Zhu contributed to the conception and design of study. Peng Mao and Baoan Wang contributed to the results discussion and paper writing. All authors participated in the preparation of the manuscript.

## Funding statement

This work was supported by the 10.13039/501100001809National Natural Science Foundation of China (81800401) and Science and Technology Development Project of Shaanxi Province (2022SF-435).

## Data availability statement

The datasets used and/or analyzed during the current study are available from the corresponding author on reasonable request.

## Limitations

The animal model involved in this study is the SD rat model of CP, which is modeled using prostate glandular injections of the complete Fuchs' adjuvant CFA. However, this method is more traditional, and the current study of CP uses C57BL/6 mice and multiple injections of prostate protein and CFA suspension for modeling, and the disease manifestation of this animal model is more closely related to the clinical study.

## Ethics approval date

The ethics approval date is 01-04-2022. The animal experiments were approved by the animal ethics committee of Shaanxi University of Traditional Chinese Medicine (SUCMDL2022401001).

## Declaration of competing interest

The authors declare the following financial interests/personal relationships which may be considered as potential competing interests:At the time the work described herein was completed, Peng Mao and Bao-An Wang were employees of Shaanxi Momentum Pharmaceutical Co., Ltd.
