# Supplementary material for: Shuangshi Tonglin capsule improves chronic prostatitis through the SIRT-1/AMPK and MAPK signalling pathways
Source: Heliyon. 2023 Nov 4;9(11):e21745. doi: 10.1016/j.heliyon.2023.e21745 (PMC10663862; doi:10.1016/j.heliyon.2023.e21745)
Supplement: Multimedia component 1 [file mmc1.pdf]

ELSEVIER

Certificate of Elsevier Language Editing Services

The following article was edited by Elsevier Language Editing Services:

"Shuangshi Tonglin capsule improves chronic prostatitis through SIRT-1/AMPK and MAPK signaling pathways"

Authored by:

Hao Wei

Date: 04-May-2022
Serial number: LE-238793-DC66121531CF
