# Supplementary material for: Shuangshi Tonglin capsule improves chronic prostatitis through the SIRT-1/AMPK and MAPK signalling pathways
Source: Heliyon. 2023 Nov 4;9(11):e21745. doi: 10.1016/j.heliyon.2023.e21745 (PMC10663862; doi:10.1016/j.heliyon.2023.e21745)
Supplement: Multimedia component 2 [file mmc2.pdf]

、

SUCMDL202204O1001

陕西中医药大学实验动物伦理委员会

动物实验伦理审查意见表

<table>
<tr><td rowspan="9">申请人填写的相关信息</td><td colspan="3">申请单位：陕西中医药大学</td></tr>
<tr><td colspan="3">申请人姓名：卫昊　　学历：硕士　　技术职称：教授</td></tr>
<tr><td colspan="3">实验名称：双石通淋胶囊通过 AMPK 信号通路改善慢性前列腺炎的作用机制</td></tr>
<tr><td colspan="3">实验目的：　科学研究</td></tr>
<tr><td rowspan="4">拟进动物情况</td><td colspan="2">动物来源：　成都达硕实验动物有限公司</td></tr>
<tr><td colspan="2">品种品系：SD 大鼠　　等级：SPF　　规格：　200±20 克</td></tr>
<tr><td>数量：72 只　（♀只；♂72 只）</td><td>申请日期：2022 年 4 月 1 日</td></tr>
<tr><td>进驻日期：2022 年 4 月 6 日</td><td>结束日期：2022 年 5 月 20 日</td></tr>
<tr><td colspan="3">实验要点，建立慢性前列腺炎动物模型，对炎症因子，抗氧化酶活性和脂质过氧化产物进行测定，检测炎症相关信号通路变化，以考察双石通淋胶囊对慢性前列腺炎的药效及作用机制。实验中采用戊巴比妥钠对动物进行麻醉,安乐处死。</td></tr>
<tr><td>项目来源</td><td>横向课题</td><td>联系电话</td><td>13992011980</td></tr>
<tr><td colspan="4">声明：我将自觉遵守实验动物伦理原则，随时接受委员会的监督与检查，如违反规定，自愿接受处罚。
项目负责人签（章）：卫昊</td></tr>
<tr><td colspan="4">伦理审查意见：☑同意　☐修改后同意　☐不同意</td></tr>
<tr><td colspan="4">审批意见
经初步审核，该项目中所涉及到的动物实验研究将遵照国际公认的动物实验室操作规范和标准操作程序，动物实验方案符合动物保护、动物福利和伦理原则，符合国家实验动物福利伦理的相关规定。
陕西中医药大学实验动物伦理委员会
（盖章）
陕西中医药大学实验动物伦理委员会</td></tr>
</table>
